# Countering the influence of tobacco

**DOI:** 10.2471/BLT.24.020424

**Published:** 2024-04-01

## Abstract

Tobacco advertising regulations need to adapt if they are to meet the challenges posed by a rapidly evolving social media and tobacco product landscape. Gary Humphreys reports.

“Coffee flavours such as cappuccino, mocha and espresso pair well with breakfast foods such as pancakes, waffles and French toast.”

These helpful hints on how to start the day feature on a web page alongside sumptuous images of the menu items in question, and are all designed to make you really want to… vape (inhale a vapour or aerosol).

Specifically, to try the coffee-flavoured vape ‘juices’ (chemical solutions of propylene glycol, glycerine, additives and, generally, nicotine) manufactured by a company based on the Isle of Man, a British Crown Dependency.

“Welcome to the world of novel tobacco product promotion,” says Becky Freeman, an expert on tobacco advertising, promotion and sponsorship (TAPS) at the University of Sydney, Australia.

The first time Freeman noticed such advertisements was in 2019. “I saw a video featuring a young woman talking about the ideal food combinations to go with Vype, an early e-cigarette brand pushed by British American Tobacco,” she says. “I remember thinking, this is like advertising before TAPS bans were introduced.”

The bans to which Freeman refers are set out in national laws implementing Article 13 of the WHO Framework Convention on Tobacco Control (WHO FCTC) – the first international treaty negotiated under the auspices of the World Health Organization (WHO) – which entered into force in 2005.

Published in 2008 as a part of guidance on implementing the treaty, Article 13 guidelines recommend a comprehensive ban on all TAPS, including smoking in films.

To date, 66 countries have implemented what are considered best-practice TAPS bans, protecting almost 2 billion people and, according to Andrew Black, team leader for development assistance at the WHO FCTC Secretariat, they have significantly decreased smoking uptake and prevalence, particularly among young people.

Unfortunately, as digital media content and the platforms designed to support their dissemination have continued to evolve, tobacco companies and other nicotine purveyors have found ways to get round the bans, notably by using social media platforms.

“Many of the bigger social media platforms and search engines have banned direct promotion, but tobacco companies have just slipped below the surface,” says Freeman. “For example, they might use Facebook web pages featuring ‘lifestyle’ content that seeks to normalize tobacco and nicotine consumption by depicting young people smoking or vaping.”

Black concurs: “Popular social media platforms have adopted policies that claim to ban tobacco advertising. However, these policies do not restrict tobacco companies from using hashtags to attract social media post attention, nor do they prevent tobacco companies from operating accounts on these platforms.”

“[Influencers] are being schooled in how to avoid posting content that looks like advertising.”

Becky Freeman

Tobacco and nicotine purveyors also amplify ‘organic’ (created spontaneously by individuals) tobacco-friendly posts by reposting, and by using influencers.

In some cases, that ‘use’ has gone beyond simply identifying and amplifying the content posted by tobacco-friendly individuals. “There are cases where social media influencers have been trained on which brands to promote, and when to post for maximum exposure,” says Freeman. “They are also being schooled in how to avoid posting content that looks like advertising.”

Freeman notes, moreover, that because the users of social media tend to be younger, tobacco and nicotine pushers are accessing precisely the demographic they need to support sustained consumption of their products.

One high-profile example of such activity is an American company using thousands of influencers to market a well-known brand of e-cigarette to teenagers in 2019.

“The JUUL example has been widely publicized, but there are others,” says Jennifer Kreslake, a researcher at Truth Initiative, a United States of America (USA)-based non-profit involved in a range of tobacco control activities, including the monitoring of TAPS on the web.

Truth Initiative uses the data they gather to report on the ways in which influencers and brand-owned accounts circumvent existing regulations, track platforms’ self-regulation efforts, and identify the scope and nature of violations to platforms’ policies.

Research published by Truth Initiative in the August 2022 issue of *Social Media and Society* revealed a possible link between tweets about new heated tobacco products posted by commercial accounts in 2016 and a surge in organic posts between 2016 and 2021.

For Kreslake, such findings should serve as a wake-up call for regulators such as the Food and Drug Administration (FDA). “FDA requirements for nicotine health warnings on advertisements and restrictions on advertising for new tobacco products must extend to influencers who endorse products on behalf of tobacco companies,” she says.

While tightening regulations might make a difference in the context of national markets, what about digital content that crosses national boundaries, as most internet content does?

“One of the big challenges we face is that content created, uploaded or broadcast in one country may be viewed in another,” admits Benn McGrady, a public health law and policies expert at WHO. “Advertisers, retailers and platforms can also be located in a different country than the country where the content is accessed, presenting opportunities for companies to engage in tobacco advertising and promotion that circumvents enforcement.”

Another challenge faced by regulators is the sheer number of companies “It’s one thing to regulate the tobacco industry where there are a few big players, but there are thousands marketing e-cigarettes,” McGrady says.

The proliferation of novel products is also a concern. As defined by the health working group of the European Parliament's committee on environment, public health and food safety, there are only three main types: tobacco-based (such as heated tobacco products); nicotine-based (i.e. aerosol products such as e-cigarettes); and nicotine pouches.

However, within each of these categories, manufacturers are constantly innovating, partly with a view to getting around TAPS regulation.

Kreslake notes that social media sites play a role in making sure consumers are aware of new products not covered by existing regulations. “When the FDA removed flavoured cartridge-based e-cigarettes from the market in 2020, the industry immediately started to promote other flavoured e-cigarette products that were still permitted,” she says.

While acknowledging the challenges faced, McGrady believes that web-based TAPS can be regulated. “The original WHO FCTC Article 13 guidelines are still applicable,” he says, underlining the fact that Article 13 calls for comprehensive bans to cover cross-border advertising, promotion and sponsorship.

McGrady believes, however, that regulation needs to be comprehensive enough to meet new challenges and have the capacity to evolve along with novel nicotine and tobacco products and TAPS strategies.

He also stresses the need for enforceability, and highlights the importance of ongoing surveillance to monitor the evolution of both online digital media platforms and novel and emerging nicotine and tobacco products.

The Truth Initiative has called upon the anti-tobacco research community to join their efforts.

However, the organization can currently obtain social media content only by scraping public-facing data and accessing application programming interfaces that social media companies consent to make available.

Some would like to oblige social media companies to share their non-public data on tobacco and novel nicotine product companies' activities.

“Currently, it is largely tobacco control stakeholders that are monitoring the amount and type of TAPS on social media platforms,” says Black. “More of this burden needs to be shifted on to the social media companies themselves.”

Freeman agrees: “The social media companies know exactly what goes through their accounts because they take a cut. They should be obliged to share that information.”

According to Freeman, dialogues between regulators and social media companies regarding disclosure are ongoing and there are clear indications of increasing pressure from regulators to bring about greater transparency and accountability.

This was reflected, for example, in the decision to adopt specific Article 13 guidelines that was taken at the recent Conference of the Parties to the FCTC.

“The guidelines address how to effectively and comprehensively ban or restrict TAPS in light of the increasing use of cross-border channels and digital media,” explains McGrady. “Governments need to implement these guidelines domestically by developing laws that cut across digital media and can be monitored and enforced.”

For Freeman, regulatory tightening cannot come too soon. “There has been this narrative that nothing can be done,” she says. “But there is a lot that government can do, as we have shown in Australia.”

Black shares her optimism: “Tobacco advertising disclosure laws, such as those that are already in place in Canada, are going to provide governments with vital information about how tobacco companies are continuing to invest in TAPS despite bans being in place,” he says.

In the coming months, attention is likely to focus on Europe, where European Commission Health Commissioner Stella Kyriakides has stated that the regulation of emerging products such as nicotine pouches are “at the core” of an ongoing evaluation of laws on tobacco products and advertising.

So, watch this cyberspace.

**Figure:**
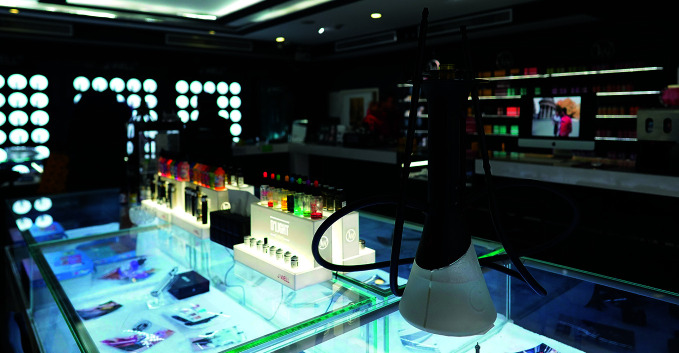
E-cigarette shop in Chaoyang District, Beijing, China

WHO/Yikun Wang

**Figure:**
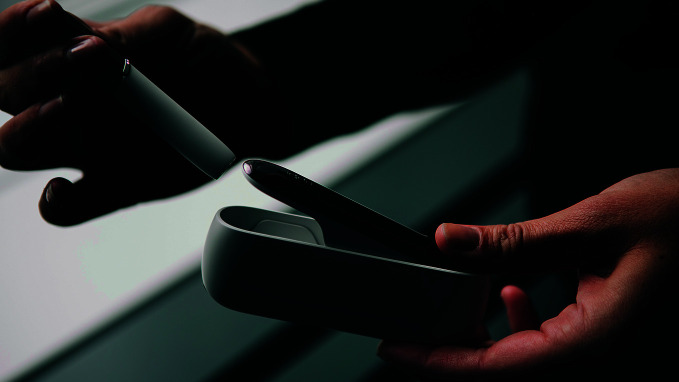
An e-cigarette container

WHO

